# Systematic characterization and biological functions of non‐coding RNAs in glioblastoma

**DOI:** 10.1111/cpr.13375

**Published:** 2022-12-01

**Authors:** Lirui Dai, Wulong Liang, Zimin Shi, Xiang Li, Shaolong Zhou, Weihua Hu, Zhuo Yang, Xinjun Wang

**Affiliations:** ^1^ Department of Neurosurgery The Fifth Affiliated Hospital of Zhengzhou University, Zhengzhou University Zhengzhou China; ^2^ Institute of Neuroscience, Zhengzhou University Zhengzhou China; ^3^ Henan International Joint Laboratory of Glioma Metabolism and Microenvironment Research Zhengzhou Henan China

## Abstract

Glioblastoma multiforme (GBM) is the most malignant and aggressive type of glioma. Non‐coding RNAs (ncRNAs) are RNAs that do not encode proteins but widely exist in eukaryotic cells. The common characteristics of these RNAs are that they can all be transcribed from the genome without being translated into proteins, thus performing biological functions, particularly microRNAs (miRNAs), long non‐coding RNAs (lncRNAs) and circular RNAs. Studies have found that ncRNAs are associated with the occurrence and development of GBM, and there is a complex regulatory network among ncRNAs, which can regulate cell proliferation, migration, apoptosis and differentiation, thus provide a basis for the development of highly specific diagnostic tools and therapeutic strategies in the future. The present review aimed to comprehensively describe the biogenesis, general features and functions of regulatory ncRNAs in GBM, and to interpret the potential biological functions of these ncRNAs in GBM as well as their impact on clinical diagnosis, treatment and prognosis and discusses the potential mechanisms of these RNA subtypes leading to cancer in order to contribute to the better design of personalized GBM therapies in the future.

## Graphical abstract

Glioblastoma multiforme (GBM) is the most malignant and aggressive glioma. Non‐coding RNAs (ncRNAs) are a category of RNAs that do not encode proteins but widely exist in eukaryotic cells. In recent years, the functions of ncRNAs, especially microRNAs (miRNAs), lncRNAs and circRNAs and their biological mechanisms of action in GBM have been studied more and more deeply, these ncRNAs may function by modifying gene transcription at the epigenetic level in *cis* or *trans* form. In this review, we comprehensively describe the biogenesis, general features, functions and mechanisms of regulatory ncRNAs, namely miRNAs, lncRNAs and circRNAs, in GBM, and interpret the potential biological functions of these non‐coding RNAs in GBM and their impact on clinical diagnosis, treatment and prognosis.

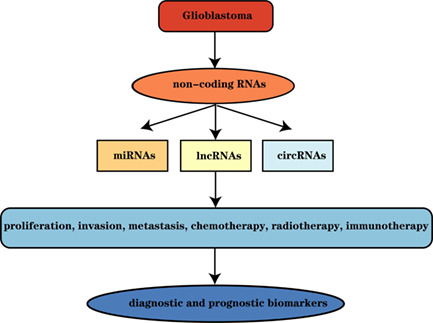

## 1 INTRODUCTION

Glioblastoma multiforme (GBM), also known as Grade IV glioma, is the most common tumour in the central nervous system (CNS) and one of the most lethal cancer types in humans, accounting for more than 30% of all CNS tumours, with a median survival time of only 12–15 months.^1^ In addition to comprehensive treatment including surgery and chemoradiotherapy, new strategies have been carried out in clinical practice; however, the median survival time of patients with GBM has not been significantly improved.^2^ This also highlights the urgent need to explore novel clinical diagnostic and treatment options for GBM, and investigate its pathogenic mechanism and functional targets to develop effective treatment and prevention measures for patients with GBM.

Non‐coding RNA (ncRNA) is a category of RNA with an extensive ability to regulate gene expression. It constitutes the majority of the transcriptome, while only ~3% of the genome contains protein‐coding RNA.^3^ The majority of ncRNAs can be transcribed as various RNA products, but do not encode proteins and are mainly responsible for gene regulation at various different levels, including pre‐transcriptional or post‐translational level.^4^ This study on ncRNAs in GBM mainly focuses on the general features and biological functions of microRNAs (miRNAs or miRs), long non‐coding RNAs (lncRNAs) and circular RNAs (circRNAs), in GBM, including cell proliferation, invasion, migration, apoptosis, angiogenesis, cell cycle, epithelial‐to‐mesenchymal transition (EMT) and changes in chemoradiotherapy sensitivity. In addition, their possible mechanism of action in the occurrence and development of GBM are discussed, which lays a foundation for the diagnosis, treatment and prognosis of patients with GBM in the future.

## 2 CLASSIFICATION AND CHARACTERISTICS OF ncRNAs

NcRNAs include various types and have several functions. NcRNAs can be divided into three types according to the length of nucleotide (nt): (i) <50 nt including miRNA, small interfering RNA and PIWI‐interacting RNA (piRNA); (ii) 50–500 nt, including ribosomal RNA (rRNA), transfer RNA (tRNA), small nuclear RNA (snRNA), small nucleolar RNA (snoRNA), SLRNA and SRPRNA; and (iii) >500 nt, including long mRNA‐like ncRNAs and lncRNAs without a poly(A) tail.^5^

According to their functions, ncRNAs can be divided into the following categories: (i) tRNAs with amino acid transport function; (ii) guide RNA with mRNA editing function; (iii) snRNAs with mRNA processing functions (cleavage and maturation); (iv) snoRNA with rRNA processing functions (cleavage and modification); (v) Telomerase RNA that has the function of DNA replication; (vi) signal recognition particles involved in protein transport and secretion; and (vii) regulation of piRNA in germ cells by combining with PIWI protein family members.^6^, ^7^ In addition, there are numerous RNAs whose functions have not been identified yet, and future research may reveal novel RNA functions. Thus, only the major ncRNAs are described in the present review.

## 3 MICRORNAs

### 3.1 Biogenesis of miRNAs

**FIGURE 1:**
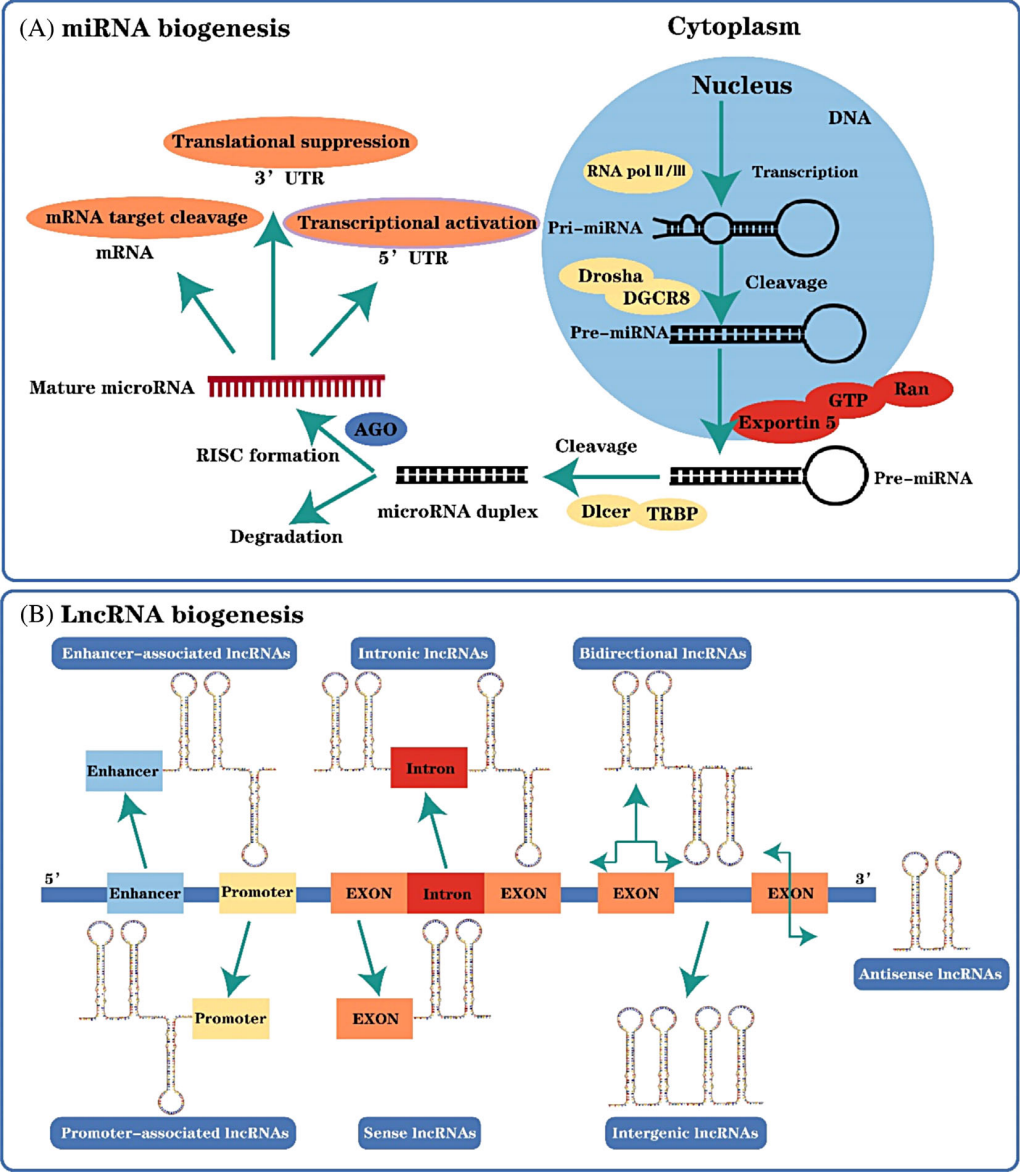
The biogenesis of microRNA (miRNA) and long non‐coding RNA (lncRNA). (A). The miRNA is preliminarily transcribed as a primary miRNA in the nucleus, then converted into a precursor miRNA under the action of Drosha and DGCR8, and transported to the cytoplasm under the action of Exportin5, GTP and Ran. After entering the cytoplasm, the precursor miRNA is transformed into double‐stranded miRNA under Dicer and TRBP processing. One miRNA is degraded, and the other miRNA becomes mature miRNA under the action of AGO and RISC, and then plays different biological roles. (B) LncRNA is transcribed by RNA polymerase II and has an mRNA‐like structure. After shearing, lncRNA has a polyA tail and promoter structure. During differentiation, lncRNA has dynamic expression and different splicing modes. (B) The different bio‐origins of lncRNAs, namely enhancer‐associated lncRNAs, intronic lncRNAs, bidirectional lncRNAs, promoter‐associated lncRNAs, sense lncRNAs and intergenic lncRNAs.

MiRNAs are endogenous ncRNAs of 18–22 nt in length that negatively regulate gene expression by interacting with the 3′ untranslated region (UTR) of mRNA targets.^8^ The biogenesis of miRNA involves multiple step: the miRNA is preliminarily transcribed as a primary miRNA under the action of RNA polymerase II/III and then converted into a precursor miRNA (pre‐miRNAs) in the nucleus.^9^, ^10^ Under the action of Exportin5, GTP and Ran, pre‐miRNAs are transferred from the nucleus to the cytoplasm, and cleaved into mature double‐stranded miRNAs.^11^ When the mature miRNAs' double helix is opened, one strand binds to the RNA‐induced silencing complex (RISC) and then binds to the target mRNA to negatively regulate gene expression, whereas the other strand is degraded^5^ (Figure 1A).

### 3.2 Functions of miRNAs

MiRNAs induce degradation at the mRNA level or translational repression by binding to mRNA transcripts in eukaryotes. MiRNAs may degrade mRNAs directly by labelling them and directing them to be degraded.^12^ MiRNA‐induced silencing complex (miRISC) is considered to mediate the degradation of mRNAs under the action of enzymes and complexes, while argonaute in miRISCs targets mRNAs by binding to miRNAs.^13^ In addition, miRNAs can reduce or inhibit gene expression by inhibiting the translation process.^14^ Beilharz et al. found in mammalian cell experiments that miRNAs can enhance translation inhibition by mediating mRNA deadenylation and decay. Numerous studies have identified that miRNAs can negatively influence the expression of target genes through atypical mechanisms. For example, Matsui et al.^15^ reported that miR‐589 could bind to the promoter RNA of cyclooxygenase‐2 (*COX2*) and cause *COX2* transcriptional activation.

### 3.3 MiRNAs in GBM

MiRNAs have been shown to play diverse roles in GBM initiation, progression and treatment, including tumour diagnosis, treatment development and optimization, and improved patient prognosis, and are critical for regulating tumour growth, metastasis, drug resistance and metabolism^16^ (Table 1).

**TABLE 1:** Summarization of the mechanism and functions of miRNAs in tumorigenesis of

MiRNAs	Role in GBM	Effect of altered expression	Target gene	References
miR‐1258	Tumour suppressor	Inhibited proliferation, therapeutic resistance, migration and invasion	E2F1	25
miR‐128	Tumour suppressor	Decreased resistance to chemotherapy	—	21
miR‐342	Tumour suppressor	Decreased resistance to chemotherapy	—	21
miR‐935	Tumour suppressor	Inhibited proliferation	FZD6	23
miR‐138	Tumour suppressor	Inhibited proliferation, increased sensitivity to chemotherapy	Survivin	137
miR‐424	Tumour suppressor	Inhibited proliferation, migration, promoted apoptosis and cell‐cycle arrest	RAF1, AKT1	49
miR‐489‐3p	Tumour suppressor	Inhibited proliferation, migration, promoted apoptosis and cell‐cycle arrest	BDNF	138
miR‐128‐3p	Tumour suppressor	Increased sensitivity to chemotherapy	RUNX1	24
miR‐138	Tumour suppressor	Inhibited proliferation, migration, promoted cell‐cycle arrest	CD44	33
miR‐370‐3p	Tumour suppressor	Increased sensitivity to temozolomide	FOXM1	28
miR‐448	Tumour suppressor	Inhibited cell viability, migration and invasion	ROCK1	139
miR‐3928	Tumour suppressor	Inhibited cell growth and invasion	MDM2, p53	32
miR‐3189	Tumour suppressor	Inhibited cell growth and promoted apoptosis	GLUT3	140
miR‐181a‐5p	Tumour suppressor	Inhibited proliferation	ADAM8	50
miR‐21‐5p	Tumour suppressor	Inhibited proliferation, migration and invasion	KANSL2	141
miR‐4286	Tumour suppressor	Inhibited invasion and mesenchymal transition	TGFB1, TGFBR2	142
miR‐674‐5p	Tumour suppressor	Inhibited proliferation and migration	Cul4b	143
miR‐185‐5p	Tumour suppressor	Inhibited proliferation and promoted apoptosis	ANXA2	144
miR‐21	Oncogene	Increased resistance to chemotherapy	—	145
miR‐221/222	Oncogene	Increased resistance to radiotherapy	—	26
miR‐133a	Oncogene	Increased proliferation, migration and invasion	TGFBR1	19
miR‐542‐3p	Oncogene	Increased proliferation and glycolytic activity	HK2	27
miR‐27a‐3p	Oncogene	Increased proliferation and resistance to temozolomide	BTG2	34
miR‐601	Oncogene	Increased proliferation	TINP1	35

Abbreviations: GBM, glioblastoma multiforme; miRNAs, microRNAs.

#### 3.3.1 MiRNAs as diagnostic and prognostic biomarkers

MiRNAs are endogenous small ncRNAs that regulate various biological functions.^17^ The majority of miRNAs have notably different expression levels between patients with GBM and normal controls. In addition, the abnormal expression of miRNAs can predict the progression of GBM, and detect the survival and prognosis of patients with GBM, which has attracted increasing attention in research. MiRNAs are expected to become biomarkers in future clinical practice.^5^

As a hotspot for cancer biomarkers, circulating miRNAs are found in cell‐free body fluids, such as serum, tissue and cerebrospinal fluid.^18^, ^19^ ParvizHamidi et al.^20^ reported that the expression of circulating *miRNA‐21* and *miRNA*‐*26a* were higher in the serum and tumour tissue samples of patients with GBM than in the normal control group, and the levels of *miRNA‐21* and *miRNA‐26a* were higher in patients with GBM before surgery than following surgery. Moreover, Wang et al. demonstrated that *miR‐128* and *miR‐342* expression in the plasma and tissue samples of patients with GBM was lower than that in normal controls, whereas their expression in patients tended to be normal after surgery and chemotherapy.^21^ These findings suggest that miRNAs can be used as GBM‐specific diagnostic biomarkers and may be helpful in the clinical treatment of patients with GBM.

The average survival time of patients with glioma is 12–15 months, the 5‐year survival rate is <5%, and the survival and prognosis of patients with GBM are worse^22^; thus, it is important to evaluate the patient's prognosis. High expression of *miR‐1258*, *miR‐935* and *miR‐128‐3p* was associated with better overall survival (OS) in patients with GBM,^23^, ^24^, ^25^ while low *miR‐542‐3p* and *miR‐221/222* clusters expression was associated with better OS in patients with GBM.^26^, ^27^ Previous studies evaluated the effect of cell‐free circulating RNA on patients with GBM. Nadaradjane et al.^28^ found that *miR‐370‐3p* could be treated in patients with GBM by enhancing temozolomide sensitivity, while it could not be used as a cell‐free circulating biomarker and was not associated with patient OS.

A number of researchers are currently developing prognostic models based on miRNA signatures. Cheng et al.^29^ developed five miRNA signatures with prognostic value for patients with GBM. Among them, risky miRNAs included *miR‐222*, *miR‐132* and *miR‐129*, whereas protective miRNAs included *miR‐145* and *miR‐20a*. High‐risk patients expressed higher levels of risky miRNAs, as well as patients with a shorter OS, whereas low‐risk controls expressed higher levels of protective miRNAs, and had a longer OS. Moreover, a prognostic model based on *miR‐125A‐5p*, *miR‐615‐5p*, *let‐7a‐5p* and *let‐7b‐5p* expression effectively predicted the correlation with OS.^30^ Santangelo et al.^31^ identified alterations in the OS and progression‐free survival (PFS) of patients with GBM upon treatment with regorafenib by using miRNAs signatures, which are models that may provide potential treatment options for patients with GBM.

#### 3.3.2 MiRNAs regulate cancer cell proliferation

The malignant proliferation of cancer cells is critical in the development of cancer. The proliferation of GBM cells is often accompanied by cell invasion, as well as oncogene activation and up‐regulation. Studies have shown that miRNAs may regulate GBM malignant proliferation by targeting multiple GBM‐related genes.

Transcription factors can enhance or inhibit gene expression by interacting with cis‐factors through their DNA‐binding domains. *MiR‐1258* can inhibit GBM cell proliferation, invasion and migration and control cell cycle by targeting transcription factor E2F1.^25^ Xu et al.^24^ indicated that *miR‐128‐3p* could act on the transcription factor RUNX to enhance the sensitivity of patients with GBM to chemotherapeutic drugs and inhibit the proliferation of tumour cells. In addition, forkhead box M1 (*FOXM1*), a member of the Forkhead Box transcription factor family, controls cell cycle processes mediated by *miR‐370‐3p*, which enhanced the sensitivity of patients with GBM to temozolomide.^28^ Moreover, *p53*, a tumour suppressor gene, forms a negative feedback loop with mouse double minute 2 homologue (*MDM2*) after targeted activation by *miR‐3928*, thus significantly inhibiting cell proliferation and invasion.^32^ *MiR‐138* can down‐regulate *CD44* expression, thus reducing the heterogeneous adhesion between tumour cells and host matrix and inhibiting the proliferation, invasion and metastasis of GBM cells.^33^

The carcinogenic role of miRNAs in GBM can also not be ignored, and its carcinogenic mechanism also needs to be further explored. *MiR‐133a*, *miR‐27a‐3p* and *miR‐601* are significantly overexpressed in GBM, and they may facilitate the proliferation of GBM cells by downregulating *TGFBR1*, *BTG* anti‐proliferation factor 2 and TGF β‐inducible nuclear protein 1.^19^, ^34^, ^35^ Previous studies have found that miR‐27a‐3p can also enhance the resistance of patients with GBM to temozolomide, which is closely associated with poor OS.^34^ In addition, Kim et al.^27^ found that *miR‐542‐3p* could induce the glycolytic activity of GBM cells by activating hexokinase 2, thus promoting cell proliferation and chemotherapy resistance, leading to reduced OS in patients.

#### 3.3.3 MiRNAs influence cell invasion and metastasis

Thanks to the in‐depth study of the aetiology and pathogenesis of GBM, it has been found that cancer‐related miRNAs have a great impact on the metastasis and invasion of GBM cells, which also provides a new strategy for improving malignant biological activities and survival in patients with GBM.^36^

EMT is markedly associated with cancer invasion and metastasis, and miRNAs affect the function of EMT in GBM, thus miRNAs may become potential diagnostic or therapeutic targets for GBM.^22^ Previous studies found that *miR‐451* inhibited the PI3K/AKT/Snail signalling pathway by activating calcium binding protein 39 in GBM, thereby inhibiting EMT and metastasis,^37^ and *miR‐200b‐3p* promoted E‐cadherin expression by down‐regulating *ERK5*, resulting in reduced invasion ability of GBM cells.^38^ Moreover, *miR‐424*,^39^ *miR‐940*,^40^ *miR‐378* ^41^ and *miR‐139‐5p* ^42^ inhibited the EMT of GBM by targeting the *KIF23*, *ZEB2*, *IRG1* and *Notch 1* genes, respectively, thus resulting in reduced invasion and metastasis of GBM cells. Furthermore, MTSS I‐BAR domain containing 1 (*MTSS1*) is important for inhibiting the proliferation and invasion of glioma cells, while *TGF‐β1* induces EMT. MiRNAs can negatively influence *MTSS1* expression, thereby facilitating the invasion and metastasis of glioma cells.^43^

*MMP2* and *MMP9* are gelatinases of the *MMP* family, which degrade extracellular matrix and are involved in tumour invasion, metastasis and immune surveillance.^44^ Overexpression of *MMP2* and *MMP9* often supported the proliferation and metastasis of GBM cells. For example, both *miRNA‐146a* and *miRNA‐564* were able to weaken the viability, invasion and migration of GBM cells by downregulating *MMP9* and *EGFR* expression.^45^, ^46^ In addition, *miR‐373* can reduce the invasion and metastasis of GBM cells by negatively influencing *HOXA* cluster antisense RNA 2, and then inhibiting vascular endothelial cadherin expression and the activity of *MMP9* and *MMP2*.^47^

MiRNAs may also affect the metastasis and invasion of GBM cells by regulating protein expression in various signalling pathways. For instance, *miR‐451* is a tumour suppressor that acts on inhibitor of NF‐κB kinase subunit β (IKKβ) to activate the NF‐κB signalling pathway and reduce *MMP9*, *MMP2*, proliferating cell nuclear antigen (*PCNA*), *cyclin D1*, *IKKβ* and phosphorylated *p65* expression, so as to weaken the proliferation and invasion of glioma cells,^48^ whereas Gheidari et al.^49^ reported that *miR‐489‐3p* could activate the PI3K/AKT signalling pathway under the action of brain‐derived neurotrophic factor and inhibit the invasion of GBM cells. The metalloproteinase integrin *ADAM* metallopeptidase domain 8 can act on the STAT3 and MAPK signalling pathways to regulate the expression of *MMP9*, *CAMP* responsive element binding protein 1, *MEK1* and *ERK2*, thus regulating the level of *miR‐181a‐5p* and promoting the invasiveness of GBM.^50^

#### 3.3.4 MiRNAs affect the sensitivity to radiotherapy and chemotherapeutic drugs

Apart from surgical treatment, radiotherapy and chemotherapy are also important means to treat GBM. However, GBM cells have been clinically shown in recent years to be resistant to radiotherapy and chemotherapy, resulting in poor prognosis of patients.^51^, ^52^ Previous studies have found that miRNAs play crucial roles in regulating the radiation and drug resistance of GBM cells.

It was found that both ionizing radiation‐induced *miR‐494* and *miR‐30 e* can activate the AKT and ERK signalling pathways by acting on *EGFR*, thereby facilitating the invasion and migration of GBM cells.^53^, ^54^ This result provides a basis for the study of radiation resistance targets in tumour radiotherapy. Areeb et al.^55^ found that EGFR expression was decreased in GBM cells resistant to radiation and temozolomide. MiRNA prediction software was used to find that *miR‐221* could negatively regulate *EGFR* and mediate resistance to radiotherapy and chemotherapy, thus becoming a potential target for GBM treatment. It has also been confirmed that the exosome‐derived *miR‐1238* of chemotherapy‐resistant GBM cells can activate *EGFR* signalling pathway by targeting *caveolin 1* and induce chemotherapy resistance in GBM cells to resist chemotherapy.^56^ In addition, *miR‐21* inhibitors in combination with paclitaxel can increase GBM cell apoptosis by inhibiting the STAT3 signalling pathway and increase sensitivity to chemotherapy drugs.^57^

#### 3.3.5 Exosomal miRNAs in GBM

Exosomes are extracellular vesicles (EVs) derived from endosomes, which are involved in intercellular communication, molecular transfer and antigen presentation.^58^ Tumour‐derived exosomes are involved in distant metastasis of tumour cells, remodelling of the tumour microenvironment and changes in drug resistance of tumour cells.^59^ Qiu et al.^60^ found that *miR‐25‐3p* exosomes can promote GBM cell proliferation, *cyclin E* expression and temozolomide resistance by targeting F‐box and WD repeat domain containing 7(*FBXW7*).^59^ It has been found that exosome *miR‐1246* isolated from cerebrospinal fluid, plasma and cells of patients with glioma promotesthe differentiation of bone marrow‐derived inhibitory cells (MDSCs) by activating the dual specificity protein phosphatase 3 (*DUSP3*)/ERK signalling pathway, while hypoxia can induce the transcription of exosomal *miR‐1246* and promote the activation of MDSCs. In addition, numerous researchers have also explored serum exosomal miRNAs of patients with GBM and normal subjects through comprehensive gene expression databases or prospective studies. For example, Yang et al.^61^ suggested that serum exosomal *miR‐98‐5p*, *miR‐183‐5p*, *miR‐323‐3p* and *miR‐19b‐3p* were potential biomarkers for GBM by using the GSE112462 and GSE122388 datasets, whereas Olioso et al.^62^ found that patients with GBM with higher exosomal miRNA expression had relatively lower OS and PFS. In conclusion, exosome‐mediated miRNAs play a variety of roles in the biological functions of GBM cells and the prognosis of patients with GBM, which requires further investigation in the future.

## 4 LONG NON‐CODING RNAs

### 4.1 Biogenesis of lncRNAs

LncRNAs have been widely studied in the field of cancer. Their length exceeds 200 nt and they do not encode proteins but retain the function of protein‐coding genes.^63^ LncRNAs are transcribed by RNA polymerase II, capped at the 5′ end and polyadenylated at the 3′ end, and most of them contain >2 exons, while >60% contain poly(A) tails.^64^ LncRNAs can be divided into sense transcript, antisense transcript, bidirectional transcript, intergenic gene, promoter, intron or enhancer according to their genome origin and distribution^65^ (Figure 1B). Although the mechanisms of lncRNAs involved in the regulation of GBM have been explored in depth recently, their biogenesis and pathogenesis remain to be further investigated due to their variety and complex mechanism.

### 4.2 Functions of lncRNAs

LncRNAs have a variety of regulatory functions, including chromatin remodelling, transcriptional and post‐transcriptional regulation, and their versatility may be the source of their functional diversity.^66^ LncRNAs can regulate gene expression by participating in responses to various stimuli through different mechanisms.^67^ It has been found that lncRNAs can form R‐loops on target gene promoters to regulate their transcription,^68^ and can also bind to transcription factors and histone modification complexes to regulate the transcription process.^69^ Previous studies have found that non‐translational lncRNAs can participate in the regulation of effector proteins expressed by genes. For example, *lncRNAs MEG3* can negatively influence *c‐Myc* expression by promoting the translation of the *PHLPP2* gene, thereby inhibiting the invasion of bladder cancer cells.^70^ Furthermore, lncRNAs participate in chromatin modification and can aggregate in the nucleus to regulate chromatin structure, or interact with chromatin modification enzymes to catalyse covalent changes of histones or nucleic acids, thus regulating genetic information.^71^ Recently, studies have found that lncRNAs can be used as miRNA sponges as competing endogenous RNAs (ceRNAs). Such LncRNAs can bind to specific binding sites of miRNAs and regulate miRNA expression and function.^72^ For example, *LINC01123* can sponge *miR‐199a‐5p* to upregulate *c‐Myc* expression in patients with non‐small cell lung cancer, thus leading to a poor prognosis.^73^ In conclusion, lncRNAs have multiple functions, and can regulate chromatin modification complexes at the transcriptional or translational level, as well as the growth, proliferation, differentiation, epigenetic inheritance and genomic imprinting of tumour cells.^74^ However, the biological functions and regulatory mechanisms of lncRNAs remain unclear to some extent, and further research is necessary.

### 4.3 LncRNAs in GBM

Increasing evidence indicates that lncRNAs can positively or negatively regulate the expression of GBM cells and the prognosis of patients with GBM. It has been found that lncRNAs play crucial roles in affecting GBM cell proliferation, apoptosis, invasion, metastasis and drug resistance to chemotherapy.^75^ The pathogenic role of lncRNAs in GBM is increasingly known, and they may even be used as potential biomarkers in the diagnosis and treatment of GBM in the future.

#### 4.3.1 LncRNAs influence cancer cell growth

The expression of *lncRNA DLGAP1* antisense *RNA* 1 (*AS1*) is higher in GBM tissues and cells than in the corresponding normal controls. Wang et al.^76^ found that silencing *lncRNA DLGAP1‐AS1* inhibited GBM cell proliferation by targeting the *miR‐515‐5p*/Rho‐associated coiled‐coil containing protein kinase 1 (*ROCK1*)/NFE2 like BZIP transcription factor 1 (*NFE2L1*) axis by functional analysis. Similarly, Zhang et al.^77^ reported that *LncRNA MIR31HG* could activate *STAT1* and promote GBM cell proliferation and inhibit cell apoptosis via the Wnt/β‐catenin signalling pathway. Previous research has found that lncRNA can promote GBM tumorigenesis through the ubiquitin‐proteasome pathway. For instance, Liang et al.^78^ demonstrated that lncRNA nuclear enriched abundant transcript 1 (*NEAT1*) deubiquitinated phosphoglycerate kinase 1 to synergistically promote GBM cell proliferation and glycolysis, whereas Lv et al.^79^ reported that lncRNA plasmacytoma variant translocation 1 (*PVT1*) could recruit *COP9* signalosome subunit 5 (*COPS5*) to deubiquitinate and stabilize tripartite motif containing 24 (*TRIM24*), thus promoting GBM cell proliferation (Table 2).

**TABLE 2:** Summarization of the cellular functions of lncRNAs in tumorigenesis of GBM.

LncRNAs	Role in GBM	Effect of altered expression	Molecular mechanism	References
DLGAP1‐AS1	Oncogene	Promoted proliferation	Regulated miR‐515‐5p/ROCK1/NFE2L1 axis	76
MIR31HG	Oncogene	Promoted proliferation and inhibited apoptosis	Activated STAT1 and Wnt/β‐catenin signals	77
NEAT1	Oncogene	Promoted proliferation and glycolysis	Deubiquitinate and stabilize PGK1	78
PVT1	Oncogene	Promoted proliferation	Deubiquitinate and stabilize TRIM24	79
LINC00998	Tumour suppressor	Inhibited proliferation	Regulated c‐Met/Akt/mTOR axis	82
SEMA3B	Tumour suppressor	Inhibited proliferation	Downregulated cyclin D1	83
RBPMS‐AS1	Tumour suppressor	Inhibited proliferation and enhanced radiosensitivity	Sponged miR‐301a‐3p/CAMTA1 axis	84
CHRM3‐AS2	Oncogene	Promoted proliferation, invasion and migration	Regulated miRNA‐370‐5p/KLF4 axis	85
MIR210HG	Oncogene	Promoted proliferation, invasion	Interaction with OCT1	86
LINC01057	Oncogene	Promoted proliferation, invasion, migration and radio‐resistance	Activated NF‐κB signals	87
PRADX	Oncogene	Promoted proliferation, basal respiration, proton leak and ATP production	Activated STAT3 signals	146
NEAT1	Oncogene	Promoted invasion and EMT	—	89
OXCT1‐AS1	Oncogene	Promoted proliferation, invasion, migration; increased the G0/G1 phase cells and decreased the G2/M phase cells	Sponged miR‐195/CDC25A axis	90
HNF1A‐AS1	Oncogene	Promoted proliferation, invasion and migration	Sponged miR‐22‐3p/ENO1 axis	91
HOXD‐AS2	Oncogene	Promoted proliferation, invasion and migration	Sponged miR‐3681‐5p/MALT1 axis	92
HOTAIRM1	Oncogene	Promoted proliferation, invasion and radiotherapy resistance	Sponged miR‐17‐5p/TGM1 axis	93
NONHSAT079852.2	Oncogene	Promoted proliferation, invasion, migration; decreased the G1 phase cells and increased the G2 phase cells	Sponged miR‐10,401‐3p/HSPA1A axis	147
MUF	Oncogene	Promoted proliferation and radiotherapy resistance, decreased apoptosis rate	Sponged miR‐34a/Snail1 axis	148
TCONS‐00004099	Oncogene	Promoted proliferation, invasion and migration	Regulated miRNA/PTPRF axis	149
LINC‐PINT	Tumour suppressor	Inhibited proliferation, invasion and migration	Regulated Wnt/β‐catenin signals	94
DGCR10	Tumour suppressor	Inhibited invasion and migration	Regulated STAT5 and NF‐κB signals	95
HRA1B	Tumour suppressor	Inhibited invasion and migration	Regulated STAT5 and NF‐κB signals	95
OIP5‐AS1	Oncogene	Promoted proliferation and temozolomide resistance	Sponged miR‐129‐5p/ IGF2BP2 axis	96
UCA1	Oncogene	Promoted proliferation and temozolomide resistance, decreased apoptosis rate	Regulated miR‐182‐5p/MGMT axis	97
LINC00511	Oncogene	Promoted temozolomide resistance	Sponged miR‐126‐5p and regulated Wnt/β‐catenin signals	98
DANCR	Oncogene	Promoted etoposide resistance	Interacted with FOXO1 and promoted FOXO2 ubiquitination	100
HOTAIR	Oncogene	Promoted proliferation, invasion and temozolomide resistance	Sponged miR‐526b‐3p/EVA1 axis	101

Abbreviations: GBM, glioblastoma multiforme; lncRNAs, long non‐coding RNAs.

Numerous studies have shown that lncRNA as ceRNA regulates tumorigenesis and promotes the progression of GBM. Wang et al.^80^ reported that *lncRNA H19* was highly expressed in GBM compared with normal controls, and *H19* promoted the proliferation and autophagy of GBM cells and inhibited apoptosis by sponging *miR‐491‐5p*. Lu et al.^81^ found that *lncRNA HAS2‐AS1* could sponge *miR‐137* to promote GBM cell and tissue proliferation and reduce survival rate (Table 3). Conversely, multiple lncRNAs also play tumour‐suppressive roles in the development of GBM. *LncRNA LINC00998* can bind to chromobox 3 (*CBX3*) to inhibit GBM cell proliferation through the c‐Met/AKT/mTOR axis and improve the survival rate of patients with GBM.^82^ In U87‐MG and U251‐MG cells, *lncRNA* semaphoring 3B (*SEMA3B*) inhibited the proliferation of *cyclin D1* by downregulating *miR‐195*.^83^ In addition, *lncRNA RBPMS‐AS1* enhanced calmodulin‐binding transcription activator (*CAMTA*) expression in GBM cells by sponging *miR‐301a‐3p*, thereby enhancing the radiosensitivity of GBM and inhibiting tumour proliferation and occurrence.^84^

**TABLE 3:** Summarization of the role of lncRNAs as ceRNA in GBM.

LncRNAs	MiRNA	Expression of mRNA	Function	References
H19	miR‐491‐5p	Upregulated ERN1	Promoted proliferation and autophagy	80
HAS2‐AS1	miR‐137	Upregulated LSP1	Promoted proliferation	81
RBPMS‐AS1	miR‐301a‐3p	Upregulated CAMTA1	Inhibited proliferation and enhanced radiosensitivity	84
OXCT1‐AS1	miR‐195	Upregulated CDC25A	Promoted proliferation, invasion, migration, induced cell cycle arrest	90
HNF1A‐AS1	miR‐22‐3p	Upregulated ENO1	Promoted proliferation, invasion and migration	91
HOXD‐AS2	miR‐3681‐5p	Upregulated MALT1	Promoted proliferation, invasion and migration	92
HOTAIRM1	miR‐17‐5p	Upregulated TGM1	Promoted proliferation, invasion and radiotherapy resistance	93
NONHSAT079852.2	miR‐10,401‐3p	Upregulated HSPA1A	Promoted proliferation, invasion, migration; decreased apoptosis rate	147
MUF	miR‐34a	Upregulated Snail1	Promoted invasion	148
OIP5‐AS1	miR‐129‐5p	Upregulated IGF2BP2	Promoted proliferation and temozolomide resistance	96
LINC00511	miR‐126‐5p	Upregulated DVL3, WISP1 and WISP2	Promoted temozolomide resistance	98
HOTAIR	miR‐526b‐3p	Upregulated EVA1	Promoted proliferation, invasion and temozolomide resistance	101

Abbreviations: ceRNA, competing endogenous RNAs; GBM, glioblastoma multiforme; lncRNAs, long non‐coding RNAs; miRNA, microRNA.

#### 4.3.2 LncRNAs regulate migration and metastasis

Increasing evidence suggests that lncRNAs promote GBM cell invasion and metastasis in vitro and in vivo. It has been demonstrated that *lncRNA CHRM3‐AS2* is upregulated in GBM, and enhances GBM cell viability while promoting GBM invasion and migration by targeting the *miRNA‐370‐5P* / KLF transcription factor 4 (*KLF4*) axis.^85^ Ho et al.^86^ found that hypoxia‐induced *lncRNA‐MIR210HG* promoted the proliferation and invasion of GBM cells by interacting with organic cation transporter 1 (*OCT1*). In addition, since a variety of lncRNAs facilitate the invasion and metastasis of GBM cells, numerous researchers have explored whether lncRNAs can maintain the mesenchymal phenotype of GBM cells. It has been found that *lncRNA LINC01057* can promote the invasion and radio‐resistance of GBM cells, as well as inducing EMT by promoting the nuclear translocation of IKKα to activate the NF‐κB signalling pathway.^87^ Yang et al.^88^ constructed EMT‐related lncRNA prognostic signatures for patients with GBM, and confirmed that EMT and metastasis‐related pathways are risk indicators for patients with GBM. Moreover, by exploiting invasion‐related lncRNAs from single‐cell *RNA* sequencing data, it was found that patients with GBM exhibiting high *NEAT1* expression had poor OS and DFS, and could promote the occurrence and progression of the malignant phenotype in patients with GBM.^89^

Previous studies have used microarray analyses to construct a ceRNA network and to explore whether lncRNAs play roles of ceRNA in GBM as well as the specific molecular mechanism. It has been reported that *lncRNA OXCT1‐AS1* competitively binds to *miR‐195* and negatively regulates *CDC25A* to facilitate the proliferation, migration and invasion of GBM cells, while the number of cells in G_0_/G_1_ phase decreases and the number of cells in G_2_/M phase increases, which promotes the malignant progression of GBM.^90^ Ma et al.^91^ found that *lncRNA HNF1A‐AS1* sponges *miR‐22* and induces its degradation to promote the malignant behaviour of GBM cells. *LncRNA HOXD‐AS2* maintains mucosa‐associated lymphoid tissue lymphoma translocation protein 1 (*MALT1*) expression by sponging *miR‐3681‐5p*, thereby inducing the proliferation, invasion and migration of GBM cells.^92^ Ahmadov et al.^93^ suggested that *lncRNA HOTAIRM1* (*HOXA* transcript antisense *RNA*, myeloid‐specific 1) upregulated transglutaminase 2 (*TGM2*) by sponging *miR‐17‐5p*, thereby upregulating the viability and invasion ability of GBM cells and enhancing their radiotherapy resistance in vitro and in vivo.

In contrast, certain studies have demonstrated that lncRNAs also act as inhibitors of cell migration. *LncRNA LINC‐PINT* (long intergenic non‐protein coding *RNA*, *p53*‐induced transcript) is downregulated in GBM tissues and cells, and plays a tumour‐suppressive role by weakening the proliferation and viability of GBM cells. Zhu et al.^94^ suggested that *LINC‐PINT* could inhibit the proliferation, invasion and EMT of GBM through the Wnt/β‐catenin signalling pathway. Huang et al.^95^ constructed a prognostic model of GBM mesenchymal associated lncRNAs, and found that DiGeorge syndrome critical region gene 10 (*DGCR10*) and *HRA1B* could significantly prevent the invasion and migration of GBM cells according to The Cancer Genome Atlas (TCGA) database (https://cancergenome.nih.gov/), and predicted that their tumour‐suppressive effect may be closely associated with the STAT5 and NF‐κB signalling pathways by Gene Set Enrichment Analysis (GSEA).

#### 4.3.3 LncRNAs affect the sensitivity to chemotherapy

Previous studies have demonstrated that Opa interacting protein 5 (OIP5)‐AS1 is a highly expressed lncRNA in GBM. Inhibition of *OIP5‐AS1* can promote the sensitivity of GBM cells to temozolomide and reduce the proliferation of tumour cells. The resistance mechanism of *OIP5‐AS1* depends on its binding to *miR‐129‐5p*, thereby upregulating insulin‐like growth factor 2 *mRNA* binding protein 2 (*IGF2BP2*) expression.^96^ In addition, upregulation of *lncRNA UCA1* contributes to temozolomide resistance in GBM, while silencing urothelial cancer associated 1 (*UCA1*) attenuates temozolomide resistance in GBM cells by inhibiting O‐6‐methylguanine‐DNA methyltransferase (*MGMT*) protein levels.^97^ It has also been found that *LINC00511* expression is increased in temozolomide‐resistant GBM cells compared with that of parental cells and is associated with a low OS rate in patients with GBM. The resistance mechanism is that *LINC00511* sponges *miR‐126‐5p* and activates the Wnt/β‐catenin signalling pathway.^98^ RNA methylation modification (m6A) is the most usual modification in eukaryotic mRNAs.^99^ *IGF2BP2* family members can recognize and stabilize target RNAs. Han et al.^100^ found that *IGF2BP2* induced differentiation antagonizing non‐protein coding RNA (*DANCR*) to interact with forkhead box protein O1 (*FOXO1*) and promote *FOXO2* ubiquitination by stabilizing *lncRNA DANCR*. Thus, the protein expression of *FOXO3* was inhibited, and the resistance of GBM cells to etoposide was eventually promoted. In addition, Wang et al.^101^ proposed that GBM serum EV‐derived *lncRNA HOTAIR* (HOX transcript antisense RNA) could induce the proliferation, invasion and temozolomide resistance of GBM cells, and its malignant characteristics were mainly induced by the upregulation of epithelial V‐like antigen 1 (*EVA1*) expression via sponging *miR‐526b‐3p*.

#### 4.3.4 LncRNAs mediate immunotherapy

In recent years, the mechanism of the immune response in GBM has been identified, and immunotherapy strategies have a potential value in initiating and enhancing host anti‐tumour immunity.^102^ However, tumour‐mediated immune suppression, including checkpoint suppression against *PD‐1/PD‐L1*, makes GBM difficult to eradicate.^103^ Yi et al.^104^ found that the novel RNA‐binding protein polymerase I and transcript release factor (*PTRF*) maintained the mRNA stability of *lncRNA NEAT1* and inhibited UBX domain protein 1 (*UBXN1*) expression, consequently activating the NF‐κB signalling pathway, promoting the binding of NF‐κB to the *PD‐L1* promoter region, and enhancing the transcription of *PD‐L1*, ultimately promoting the immune evasion of GBM cells. Previous studies focused on the transcriptome‐wide m^6^A methylation profile of lncRNAs in GBM. It was suggested that heat shock 70 kDa protein 7 (*HSPA7*) could be a possible prognostic risk factor for patients with GBM according to the analysis of GBM m^6^A sequencing data, and the results revealed that *HSPA7* could upregulate yes‐associated protein 1 (*YAP1*) and lysyl oxidase (*LOX*) expression in GBM stem cells, thus promoting the recruitment of macrophages into the tumour microenvironment. However, silencing *HSPA7* could inhibit the efficiency of *PD‐1* therapy, making it possible to use *HSPA7* as a new target for immunotherapy.^105^ In addition, GBM data based on lncRNA high‐throughput sequencing obtained from TCGA database are currently being explored by various researchers. Li et al.^106^ identified immune‐related lncRNAs by Pearson correlation and constructed an immune‐lncRNAs co‐expression network. A total of five immune‐related lncRNA signatures were obtained, and it was verified that the high‐risk group had lower OS and worse overall prognosis. Moreover, via GSEA, Gao et al.^107^ analysed the transcriptome information of 144 GBM cases using TCGA database, and evaluated and identified six lncRNAs associated with immunophenotypes, including *USP30‐AS1*, *LINC01684*, *PSMB8‐as1*, *AL133264.2*, *HCP5* and *LINC01506*. Reverse transcription‐quantitative PCR analyses confirmed that these six lncRNAs were highly expressed in GBM tissues compared with their expression in normal samples, and were crucial in tumour immune infiltration; thus, they may become diagnostic indicators of the GBM immunophenotype in the future. In conclusion, lncRNA‐dependent immune regulation and immune‐related lncRNAs in the immunophenotype and treatment strategies of GBM are promising research hotspot.

#### 4.3.5 LncRNAs may serve as promising prognostic and diagnostic biomarkers

Due to the stable secondary structure of lncRNA, various studies have included it as a promising peripheral biomarker and prognostic indicator for GBM.^108^ It has been found that the mRNA expression of *lncRNA HOTAIR* in the serum of patients with GBM is higher than that of low‐grade gliomas controls. Tan et al.^109^ evaluated the serum *HOTAIR* level of patients with GBM and controls as a potential diagnostic marker, with an AUC (area under the curve) value of 0.913, and a sensitivity and specificity of 86.1% and 87.5%, respectively. Shen et al.^110^ observed that *HOTAIR* drove the proliferation and invasion of GBM cells by analysing the serum of 106 patients with GBM, and *HOTAIR* expression was negatively correlated with the OS of patients, thus becoming an indicator of poor prognosis in GBM. In contrast, growth arrest specific 5 expression in GBM serum was lower than that in normal controls, and its downregulation was associated with decreased disease‐free survival and OS. In general, this type of minimally invasive liquid biopsy is expected to greatly improve the diagnosis and prognosis of patients with GBM. Wang et al.^111^ proposed an exosome‐derived ceRNA network based on the Gene Expression Omnibus and TCGA databases by LASSO and multivariate Cox regression analysis, and recognized *HOTAIR*, SRY‐box transcription factor 21‐AS1 and six‐transmembrane epithelial antigen of prostate 3‐AS1 as possible prognostic exosomal lncRNAs. The nomogram was constructed based on the patients' age, isocitrate dehydrogenase status, MGMT promoter status, selection of chemoradiotherapy, and exosomal lncRNA, and the results showed that it had good recognition and prediction ability. In conclusion, increasing evidence has shown that lncRNA can be applied as possible diagnostic and therapeutic biomarkers for GBM, which plays important roles in detecting the effect of therapy and tumour recurrence.

## 5 CIRCULAR RNAs

### 5.1 Biogenesis of circRNAs

**FIGURE 2:**
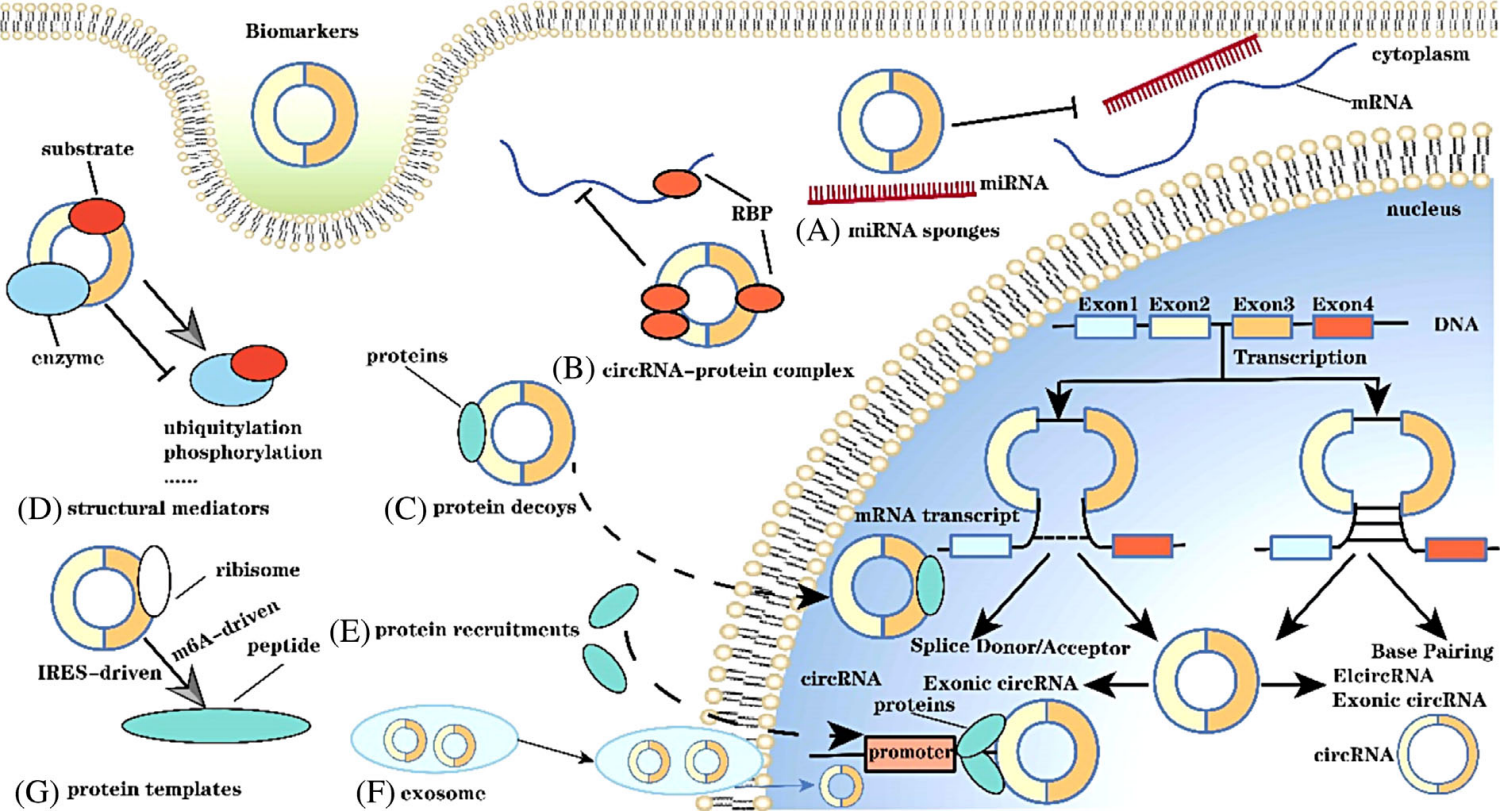
The biogenesis and biological functions of circular RNAs (circRNAs). (1) LARIAT‐driven circularization: the cleaved donor at the 3′ end of Exon 1 binds covalently to the cleaved acceptor at the 5′ end of Exon 4. Lariat is generated under exon hopping, and then the intron is removed to form circRNA. (2) Intron pairing‐driven circularization: successive introns form a circular structure, which is then cleaved to form exon circRNA. (A–G) systematically summarizes the general functions of circRNA.

CircRNAs were initially found in *RNA* viruses by electron microscopy by Sanger et al.^112^ In 1993, the structure of circRNAs was confirmed.^113^ CircRNA is biosynthesized in the presence of precursor RNA transcribed by RNA polymerase II and RNA‐binding protein,^114^ it can be divided into intronic circRNAs, exonic circRNAs and EciRNA.^115^ The mechanism of circRNA maturation is not fully understood. Jeck et al.^116^ have proposed three circRNA formation models, namely intron‐pairing‐driven circularization, RNA‐binding protein‐dependent circularization and lariat‐driven circularization. (Figure 2).

### 5.2 Functions of circRNAs

The most common biological function of circRNAs is miRNA sponge (Figure 2), which means that the circRNA acts as a competitive inhibitor to competitively inhibit the binding between miRNA and target genes, so as to protect mRNA from miRNA degradation and maintain the stability of target genes.^117^ For instance, *circENTPD7* affects *ROS1* expression by sponging *miR‐101‐3p*, thus facilitating the proliferation of GBM cells and enhancing cell viability.^117^ Zhang et al. found that *circFOXO3* acts as a ceRNA to upregulate the nuclear factor expression of nuclear factor of activated T cells 5 (*NFAT5*) by sponging *miR‐138‐5p* and *miR‐432‐5p*, so as to promote tumour invasion and metastasis.^118^ CircRNA can bind to RNA‐binding proteins to influence the expression of target genes, and it can also inhibit the binding of miRNA and RNA‐binding proteins to indirectly regulate the function of RNA‐binding proteins.^119^ In addition, circRNA can induce the transfer of target proteins to the nucleus to perform biological functions. It was found that *circ‐AMOTL1* (angiomotin like 1) increased the expression and stability of *c‐Myc* in the nucleus by interacting with *c‐Myc* and *circ‐AMOTL1* expression also enhanced the binding affinity of *c‐Myc* to multiple promoters.^120^ Moreover, circRNA can promote the interaction between certain enzymes and substrates and influence the kinetics of reaction, such as ubiquitination and phosphorylation.^121^ Since circRNA is mainly localized in the nucleus, it can recruit proteins to promoters or other specific sites at the transcriptional level, thereby enhancing the function of ribonucleoprotein and regulating gene expression.^122^ Certain circRNAs can also be carried by exosomes to participate in functional interactions between cells. Finally, circRNA enters the inteRNAl ribosome entry site (IRES) and m6A sites via inteRNAl ribosomes in the 5′ UTR of mRNA to initiate the translation process, IRES initiates the circRNA translation process by recruiting ribosomes, whereas m6A initiates the translation process by binding to eukaryotic initiation factor 3 (*EIF*).^123^ In conclusion, circRNA has a variety of functions, among which, the most often studied and reported is miRNA sponge, including in GBM. The findings of future studies will suggest new possibilities for the future diagnosis and treatment of GBM by circRNA.

### 5.3 CircRNAs in GBM

**FIGURE 3:**
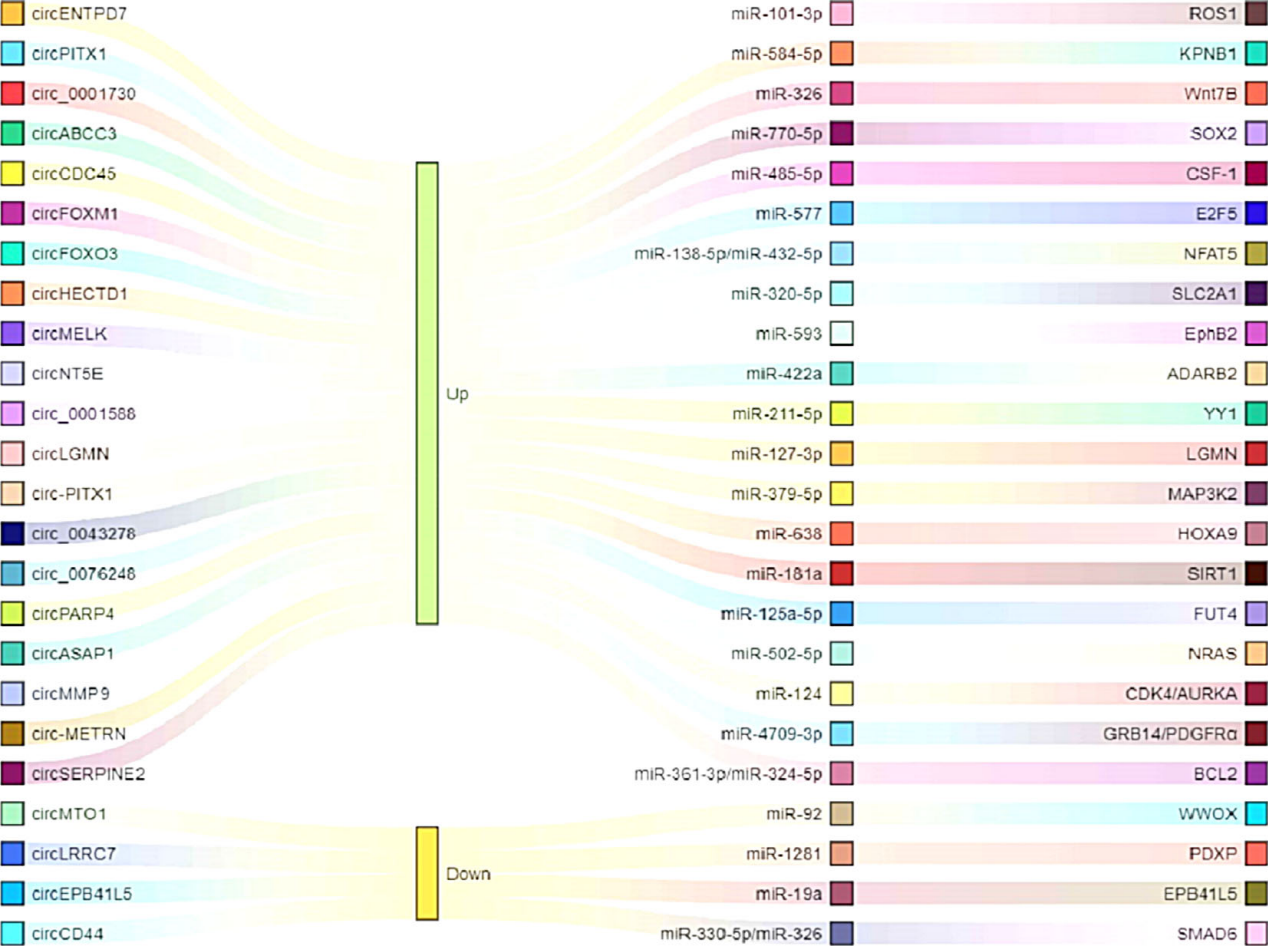
Some important circular RNAs (circRNAs) as microRNA (miRNA) sponges and corresponding target genes. circRNAs can be served as miRNAs sponge, thus targeting target genes, which positively or negatively participate in the interaction or crosstalk of various key pathways in glioblastoma, thus affecting the biological function of glioblastoma cells.

Increasing evidence considers circRNA as a new hotspot for the study of the association between ncRNAs and various cancer types. The mechanism of circRNAs in cancer, particularly in GBM, is still being explored. To date, the research on circRNAs in GBM has mainly focused on ‘miRNA sponges’, which refers to the participation of circRNA in the progression of GBM as a ceRNA, which can bind to downstream target genes of miRNA, thereby inhibiting the binding of miRNA and target mRNA, thus protecting mRNA from miRNA degradation.^117^ Furthermore, numerous studies have suggested that circRNAs may have a potential role in the diagnosis and prognosis of GBM. The present review has summarized the oncogenic or anticancer effects of circRNAs as miRNA sponges in GBM and their mechanism of action (Figure 3).

#### 5.3.1 CircRNAs as tumour promoters

CircRNAs, as miRNA sponges, can promote numerous malignant behaviours in GBM tissues and cells, such as promoting tumour cell proliferation and sensitivity to chemotherapy drugs. It was found that *circ‐MELK* was upregulated in GBM, and its high expression promoted the proliferation and viability of GBM cells, while knockdown of *circ‐MELK* could significantly inhibit GBM growth in vitro and in vivo. A previous study indicated that *circ‐MELK* could promote the mesenchymal transition of GBM cells and maintain the roles of tumour stem cells by targeting EPH receptor B2 (*EphB2*) via sponging *miR‐593*.^124^ *CircNT5E* (circ 5′‐nucleotidase ecto [*NT5E*]) derived from *NT5E* is formed by binding of adenosine deaminase RNA‐specific B2 (*ADARB2*) binding to the flanking site of the circRNA intron, and its high expression often causes the proliferation and distant metastasis of GBM cells in GBM. *CircNT5E* can restrain cell proliferation, invasion and migration by targeting *PIK3CA* and *NT5E* via sponging *miR‐422a* in GBM.^125^ In addition, *circ‐LGMN* (legumain) may also promote the development of GBM by targeting the *miR‐127‐3p /LGMN* axis, and *circ‐LGMN* can upregulate *LGMN* expression by sponging *miR‐127‐3p*, thus facilitating the proliferation and invasion of tumour cells and tissues.^126^ Wei et al.^127^ also found that *EIF4A3*‐induced *circASAP1* (ArfGAP with SH3 domain, ankyrin repeat and PH domain 1) was markedly upregulated in recurrent GBM tissues and temozolomide‐resistant cell lines, and *circASAP1* upregulated *NRAS* expression by sponging *miR‐502‐5p*, increasing the proliferation of temozolomide‐resistant GBM cells and their resistance to temozolomide.

Previous studies indicated that circRNAs are important for GBM invasion, metastasis, and radio‐sensitization. It has been found that *circFOXO3* expression is upregulated in GBM tissues. Functional experiments have found that *circFOXO3* significantly enhances the invasion and migration of GBM cells, and biochemical analysis has shown that *circFOXO3*, as a ceRNA, can upregulate the expression of the nuclear factor of *NFAT5* by sponging miR‐138‐59 and *miR‐432‐5p*, thus promoting the progression of GBM in vitro and in vivo.^128^ Activated *EGFR* signalling drives the occurrence and progression of the majority of GBM tumours, and the secreted E‐cadherin protein variant encoded by E‐cadherin‐RNA plays an important role in activating *EGFR* signal transduction in GBM and promoting tumorigenicity of glioma stem cells.^129^ Wang et al.^130^ indicated that *circMMP9* upregulated the protein expression of *CDK4* and aurora kinase A (*AURKA*) by targeting *miR‐124*, and facilitated the proliferation, invasion and migration of GBM cells via the *circMMP9/ miR‐124* axis. It has been previously found that low‐dose radiation‐induced exosomes (*ldrEXOs*) and ldrEXOs‐derived *circ‐METRN* (meteorin) affect the progression and radiotherapy sensitivity of GBM. Low‐dose radiation can increase the level of *circ‐METRN* by stimulating *IdrEXOs* secretion, thereby increasing the level of the DNA damage repair protein *γ‐H2AX*, and facilitating the proliferation, invasion, migration and radiation resistance of GBM cells by targeting growth factor receptor‐bound protein 14 (*GRB14*)/platelet‐derived growth factor receptor (*PDGFRα*) with *miR‐4709‐3p*.^131^

#### 5.3.2 CircRNAs as tumour suppressors

Although circRNAs are usually involved in gene regulation, tissue and cell carcinogenesis and other pathophysiological processes associated with high expression levels in GBM, certain circRNAs also play tumour suppressor roles in GBM. It was found that *circ‐SHPRH* (SNF2 histone linker PHD RING helicase) was downregulated in GBM but highly expressed in normal human brain, and *SHPRH‐146AA* increased the ability of *SHPRH* to ubiquitinate *PCNA* by protecting *SHPRF* from ubiquitin proteasome degradation, resulting in decreased tumour proliferation and reduced tumour‐genic ability.^132^ *CircAKT3* is an AKT transcriptional variant, and its expression level in GBM tissues is markedly lower than that in adjacent normal controls. *AKT3‐174aa* encoded by *circAKT3* negatively regulates the PI3K/AKT signalling pathway by interacting with phosphorylated pyruvate dehydrogenase kinase 1 (*PDK1*) in GBM; thus, the proliferation, radiation resistance and tumorigenic ability of GBM cells could be inhibited, which would provide benefits for the long‐term prognosis of patients.^133^ Lou et al.^134^ found that the protein expression of *circCDR1* (cerebellar degeneration‐related protein 1) was notably reduced in GBM, and may serve as a reliable predictor of OS in patients with GBM. Previous studies have found that *circCDR1* as stabilizes *p53* protein by de‐ubiquitination of *p53*, thus making *p53* interact with the DNA‐binding domain, disrupting the formation of the *p53/MDM2* complex, and finally protecting GBM cells from DNA damage, thus playing a tumour suppressor role. Yang et al.^135^ found that the open reading frame of *circ‐FBXW7* encodes a small functional protein *FBXW7‐185aa* driven by the ribosome entry site. The expression levels of *circ‐FBXW7* and *FBXW7‐185aa* were low in GBM, and the up‐regulation of *CIRC‐FBXW7* and *FBXW7‐185aa* could significantly inhibit cell proliferation and cell cycle accelerating, and improve the OS of GBM patients, thus becoming a potential functional protein with prognostic significance in GBM. In addition, it has been suggested that *circCD44* can regulate *SMAD6* expression by sponging *miR‐326* and *miR‐330‐5p*, thereby downregulating *circCD44* and causing reduced proliferation, invasion and migration ability of GBM cells.^136^ In conclusion, the mechanism of the tumour‐suppressive effect of circRNA in GBM remains unclear; thus, it is necessary to further explore its specific function and mechanism in GBM in the future (Table 4).

**TABLE 4:** Mechanism and functions of circRNAs in GBM tumorigenesis.

CircRNAs	Role in GBM	Cancer phenotype	Sponge miRNAs	References
circENTPD7	Oncogene	Promoted proliferation and viability	miR‐101‐3p	118
circPITX1	Oncogene	Promoted proliferation, migration, invasion, angiogenesis and induced cell cycle arrest	miR‐584‐5p	150
circ_0001730	Oncogene	Promoted proliferation, invasion and EMT	miR‐326	151
circABCC3	Oncogene	Promoted proliferation, migration, invasion, angiogenesis and apoptosis	miR‐770‐5p	152
circCDC45	Oncogene	Promoted proliferation, migration and invasion	miR‐485‐5p	153
circFLNA	Oncogene	Promoted proliferation, and invasion	miR‐199‐3p	154
circFOXM1	Oncogene	Promoted proliferation, migration and invasion	miR‐577	155
circFOXO3	Oncogene	Promoted migration and invasion	miR‐138‐5p/miR‐432‐5p	128
circHECTD1	Oncogene	Promoted proliferation and migration	miR‐320‐5p	156
circMELK	Oncogene	Promoted proliferation, and invasion	miR‐593	124
circNT5E	Oncogene	Promoted proliferation, migration and invasion	miR‐422a	125
circNUP98	Oncogene	Promoted proliferation	miR‐519a‐3p	157
circSKA3	Oncogene	Promoted proliferation	miR‐1	158
circ_0001588	Oncogene	Promoted proliferation, migration and invasion	miR‐211‐5p	159
circLGMN	Oncogene	Promoted proliferation and migration	miR‐127‐3p	126
circNF1	Oncogene	Promoted proliferation	miR‐340	160
circ‐PITX1	Oncogene	Promoted proliferation and apoptosis	miR‐379–5p	161
circ_0043278	Oncogene	Promoted migration and invasion	miR‐638	162
circ_0076248	Oncogene	Promoted proliferation and induced cell cycle arrest	miR‐181a	163
circPARP4	Oncogene	Promoted proliferation, migration invasion and EMT	miR‐125a‐5p	164
circASAP1	Oncogene	Promoted proliferation and TMZ resistance	miR‐502‐5p	127
circMMP9	Oncogene	Promoted proliferation, migration and invasion	miR‐124	130
circ‐METRN	Oncogene	Promoted proliferation, migration, invasion and apoptosis	miR‐4709‐3p	131
circSERPINE2	Oncogene	Promoted proliferation and apoptosis	miR‐361‐3p/miR‐324‐5p	165
circMTO1	Tumour suppressor	Inhibited proliferation,	miR‐92	166
circ‐EPB41L5	Tumour suppressor	Inhibited proliferation, migration and invasion	miR‐19a	167
circCD44	Tumour suppressor	Inhibited proliferation, migration and invasion	miR‐330‐5p/miR‐326	136

Abbreviations: circRNA, circular RNA; GBM, glioblastoma multiforme; miRNA, microRNA.

## 6 CONCLUSION

In conclusion, ncRNAs play important roles in the occurrence and development of GBM. The present review summarized the roles of three typical regulatory ncRNAs, as well as their functions and mechanisms in GBM as shown in Tables 1, 2, 3, 4. To date, the function and mechanism of miRNAs and lncRNAs in GBM have been widely studied and consensus is gradually being reached among researchers. However, the mechanism of action of ncRNAs, including circRNAs, remains unclear. Future studies should provide further information on the association between ncRNAs and GBM, which may help to improve the clinical treatment and long‐term prognosis of GBM.

## AUTHOR CONTRIBUTIONS

Lirui Durai wrote the article. Wulong Liang, Shaolong Zhou, Zimin Shi, Xiang Li, Weihua Hu and Zhou Yang revised it critically for important intellectual content and gave important advice. Xinjun Wang provided the overall idea of the article and revised the original article. All authors read and approved the article and agree to be accountable for all aspects of the research in ensuring that the accuracy or integrity of any part of the work are appropriately investigated and resolved.

## FUNDING INFORMATION

This work was supported by the National Natural Science Foundation of China under Grant number (81972361 to Xinjun Wang) and Medical science and Technology project of Henan Province under Grant number (LHGJ20210487 to Wulong Liang). Wulong Liang revised the article critically for important intellectual content and gave important advice and Xinjun Wang provided the overall idea of the article and revised the original article.

## CONFLICT OF INTEREST

The authors declare that there is no conflict of interest that could be perceived as prejudicing the impartiality of the research reported.

## Data Availability

The data that support the findings of this study are available from the corresponding author upon reasonable request.
